# The Extracellular Matrix and the Immune System in Acute Lung Injury: Partners in Damage and Repair

**DOI:** 10.3390/biomedicines14010057

**Published:** 2025-12-26

**Authors:** Feiyan Xie, Yuheng Sun, Jing Wang, Wei Luo, Xinxin Zhang, Yusi Cheng, Jie Chao

**Affiliations:** Jiangsu Provincial Key Laboratory of Critical Care Medicine, Zhongda Hospital, Department of Physiology, School of Medicine, Southeast University, Nanjing 210009, China

**Keywords:** acute lung injury (ALI), extracellular matrix (ECM), immune system, inflammatory microenvironment

## Abstract

Acute lung injury (ALI) is driven by a complex interplay between immune dysregulation and structural matrix remodeling. Although inflammation, oxidative stress, and disturbances in the coagulation–fibrinolysis system have long been recognized as core pathogenic drivers, growing evidence demonstrates that the extracellular matrix (ECM) functions as an active regulator of lung injury and repair rather than a passive structural scaffold. This review synthesizes current advances in ECM biology and immunopathology to delineate how ECM remodeling influences, and is concurrently shaped by, the inflammatory microenvironment. We outline how biochemical and physical modes of ECM remodeling engage in bidirectional crosstalk with the immune system. Emerging therapeutic strategies targeting this ECM–immune axis are critically evaluated, including modulation of protease activity, interventions that reprogram cell–matrix interactions, and approaches that restore ECM integrity using stem cells or engineered biomaterials. By redefining ALI as a disease of immune–matrix reciprocity, this review underscores the ECM as both a structural framework and a dynamic immunoregulatory hub, providing conceptual and mechanistic insights that may guide the development of precision therapies for ALI and related pulmonary disorders.

## 1. Introduction

Acute lung injury (ALI) and its more severe manifestation, the acute respiratory distress syndrome (ARDS), are heterogeneous clinical syndromes characterized by diffuse alveolar damage and severe impairment of gas exchange [1]. Although sepsis is a common precipitating condition in critically ill patients [2,3], ALI and ARDS may arise from a wide spectrum of direct and indirect insults to the lung. Direct pulmonary causes include bacterial or viral pneumonia, aspiration of gastric contents, lung or chest trauma, pulmonary hemorrhage, drowning, and inhalation of smoke or toxic gases. Indirect, extrathoracic causes originate outside the lung and include sepsis, transfusion-related lung injury, drug toxicity, severe non-thoracic trauma, disseminated intravascular coagulation, and acute pancreatitis [3,4,5]. In addition, ALI may develop secondary to other clinical conditions such as aspiration during anesthesia, burn-related infections, fat embolism, heat stroke, and prolonged exposure to environmental pollutants [3], underscoring the mechanistic heterogeneity and clinical breadth of this syndrome.

Irrespective of the initiating cause, ALI is defined by injury to both alveolar epithelial cells and pulmonary capillary endothelial cells, resulting in disruption of the alveolar capillary barrier, increased vascular permeability, and the development of noncardiogenic pulmonary edema. The relative contribution of epithelial and endothelial injury varies according to etiology, with direct pulmonary insults primarily targeting the alveolar epithelium, whereas indirect forms of ALI, particularly those associated with sepsis, are dominated by endothelial activation, systemic inflammation, and microvascular dysfunction. Despite these etiological differences, diverse forms of ALI converge on shared pathological pathways involving dysregulated inflammation, oxidative stress, and abnormalities in coagulation and fibrinolysis [3,6]. The resulting reductions in lung volume and compliance, together with severe ventilation–perfusion mismatch, manifest clinically as refractory hypoxemia and progressive respiratory failure [7] and may evolve into ARDS, a condition associated with high mortality, prolonged intensive care unit admission, and substantial long-term morbidity [8,9,10].

In contemporary clinical practice, the physiological abnormalities of ALI and ARDS are contextualized within standardized diagnostic frameworks. Early definitions culminated in the American–European Consensus Conference (AECC) criteria, which introduced the arterial oxygen tension to fraction of inspired oxygen ratio (PaO_2_/FiO_2_) as a central physiological parameter, defining ALI by a PaO_2_/FiO_2_ ratio of less than 300 mmHg and ARDS as its more severe subset with a ratio of less than 200 mmHg [11,12,13]. This framework was subsequently refined by the Berlin definition, which reclassified ARDS into mild, moderate, and severe categories based primarily on the degree of hypoxemia, with PaO_2_/FiO_2_ thresholds of 200–300 mmHg, 100–200 mmHg, and ≤100 mmHg, respectively, assessed under standardized ventilatory conditions including a minimum level of positive end-expiratory pressure (PEEP) or continuous positive airway pressure (CPAP) of 5 cm H_2_O [14,15]. The Berlin definition further integrates bilateral pulmonary opacities consistent with noncardiogenic pulmonary edema as an imaging criterion, thereby unifying physiological severity with structural lung injury [9]. These severity strata are associated with stepwise increases in mortality and duration of mechanical ventilation [16,17,18,19,20], underscoring the prognostic relevance of hypoxemia-based classification.

Most recently, the 2023 global definition of ARDS has been proposed to enhance early recognition. A major shift is that it applies to patients receiving any form of respiratory support, removing the previous Berlin requirement for a specific PEEP level. It broadens the criteria to include patients on high-flow nasal oxygen (HFNO) at ≥30 L/min. For hypoxemia assessment, it retains a PaO_2_/FiO_2_ threshold of ≤300 mmHg but alternatively accepts an SpO_2_/FiO_2_ ratio of ≤315 when arterial blood gas analysis is unavailable (and SpO_2_ is ≤97%). Regarding imaging, while chest radiograph or CT remains standard, lung ultrasound is now endorsed as a validated alternative when standard imaging is not accessible, increasing utility in resource-limited settings [21,22,23].

Within this refined clinical continuum, ALI/ARDS are increasingly recognized as dynamic disease states in which progressive epithelial and endothelial injury, inflammatory amplification, and extracellular matrix remodeling evolve in parallel. Importantly, expanded diagnostic criteria that enable earlier and less resource-dependent identification highlight that many extracellular matrix (ECM)–immune interactions are initiated before the onset of severe hypoxemia or established fibrotic remodeling. Recognition of these early, predominantly exudative and inflammatory stages is therefore critical for defining when matrix-driven inflammatory signaling is most active and potentially most amenable to therapeutic intervention [21].

Traditionally regarded as a passive structural scaffold, the ECM is now recognized as a dynamic and instructive component of the pulmonary microenvironment that actively regulates cellular behavior, immune responses, and tissue repair [24,25,26,27,28,29,30,31]. These matrix alterations are accompanied by profound changes in the ultrastructure of the alveolar septa, which form the histopathological basis of impaired gas exchange. In the lung, pathological remodeling of the ECM is not merely a consequence of injury but also a driver of sustained inflammation and aberrant healing. However, most current therapeutic strategies in ALI continue to focus predominantly on soluble inflammatory mediators [6], largely overlooking the role of the solid phase ECM microenvironment in shaping immune cell function.

Conceptualizing ALI as a disorder of immune–matrix reciprocity therefore provides a unifying framework that integrates structural pathology with immune dysregulation and repair processes, and highlights the ECM as a critical, yet underappreciated, regulator of lung injury and resolution. This review synthesizes current advances to delineate how biochemical and physical modes of ECM remodeling intersect with the immune pathways, shaping the onset, progression, and outcome of ALI. By integrating clinical staging with molecular and biophysical matrix remodeling, this framework aligns pathological timing with therapeutic opportunity. Understanding this multidirectional exchange may guide the development of targeted therapies that modulate the ECM–immune axis to restore lung homeostasis.

## 2. Structural and Histopathological Hallmarks of Acute Lung Injury

### 2.1. The Alveolar Septum and ECM in the Healthy Lung

The defining pathological feature of ALI is diffuse alveolar damage, which reflects profound disruption of the normal architecture of the alveolar septa [6,32]. In the healthy lung, gas exchange occurs across an exquisitely thin alveolar septum (or blood–air barrier). This barrier is formed by a continuous alveolar epithelium and a continuous capillary endothelium, which are closely apposed and structurally integrated by the ECM. The ECM within the septum is heterogeneous: basement membranes are specialized, dense layers that underlie and anchor the epithelial and endothelial cells, while the interstitial matrix occupies the space between these two basement membranes, providing elastic recoil [24,33,34].

Under physiological conditions, the alveolar basement membrane is enriched in laminins, type IV collagen, nidogen, and heparan sulfate proteoglycans (HSPGs), forming a dense, highly organized network that anchors epithelial and endothelial cells, maintains barrier integrity, and transduces homeostatic biochemical and mechanical signals. In contrast, the interstitial matrix is relatively sparse and compliant, composed primarily of elastin fibers interwoven with fibrillar collagens, providing elasticity and recoil essential for normal lung mechanics. Together, these matrix components maintain alveolar stability while permitting rapid deformation during respiration [35,36,37,38]. Importantly, the diverse clinical triggers of ALI converge on the ECM through distinct but overlapping injury paradigms. Direct pulmonary insults such as pneumonia and aspiration primarily initiate epithelial basement membrane disruption, whereas indirect systemic insults, including sepsis and transfusion-related lung injury, predominantly drive endothelial activation, plasma protein leakage, and fibrin-rich provisional matrix formation, together shaping the trajectory and pattern of ECM remodeling.

### 2.2. Compositional Shift of the ECM in ALI

In ALI, this finely tuned structural organization is profoundly disrupted. Early injury to alveolar epithelial and capillary endothelial cells leads to breakdown of the basement membrane, loss of epithelial anchorage, and increased vascular permeability [39,40,41]. One of the hallmark compositional shifts that defines ALI pathology is the degradation and loss of homeostatic matrix components, including laminin and elastin, accompanied by the accumulation of a provisional ECM rich in fibrin and fibronectin [40,42,43,44]. This provisional matrix originates largely from leaked plasma proteins, particularly fibrinogen, which extravasate into the alveolar space and are subsequently converted to fibrin [45,46]. Fibronectin, derived from both plasma and locally activated mesenchymal cells, further stabilizes this matrix and provides adhesive ligands for inflammatory cells.

### 2.3. Hyaline Membranes as Active Structural and Inflammatory Entities

A defining histopathological manifestation of ALI is the formation of hyaline membranes along the alveolar walls [47,48]. These structures are not inert deposits but represent active precipitates composed of fibrin polymers, plasma proteins, necrotic epithelial debris, and surfactant remnants [49]. Hyaline membranes physically line and occlude alveolar surfaces, markedly increasing diffusion distance and mechanically impairing gas exchange [50,51]. In addition to their barrier effects, these fibrin-rich matrices provide a bioactive scaffold that concentrates cytokines, chemokines, and damage-associated molecular patterns, thereby amplifying local inflammation and leukocyte retention [52].

As ALI evolves, persistent provisional matrix deposition within the alveolar and interstitial compartments further alters lung mechanics by increasing tissue stiffness and disrupting normal alveolar architecture. The transition from a laminin and elastin-dominated homeostatic matrix to a fibrin and fibronectin-rich inflammatory matrix, therefore, represents a central structural hallmark of ALI. This compositional shift not only reflects tissue injury but also actively shapes immune cell behavior, inflammatory persistence, and the balance between effective repair and pathological remodeling. Establishing this structural baseline is essential for interpreting how immune-mediated signaling intersects with ECM remodeling to drive both acute tissue damage and divergent repair trajectories in ALI.

## 3. Temporal Landscape of ECM Remodeling in ALI

### 3.1. Classical ALI Staging Revisited in the Context of ECM Remodeling

**Figure 1:**
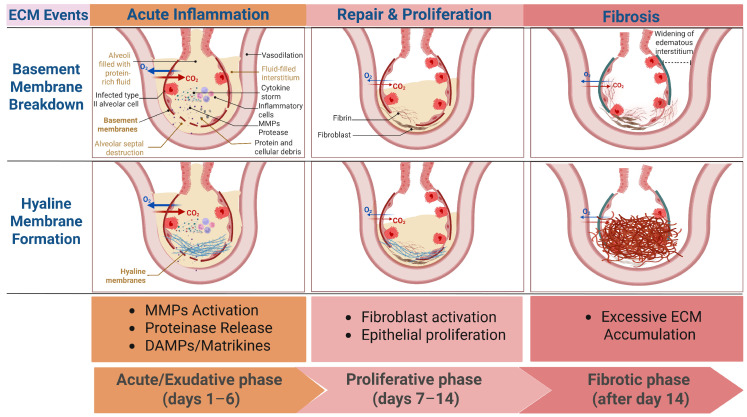
Temporal dynamics of extracellular matrix (ECM) remodeling across the clinical phases of acute lung injure (ALI). This schematic illustrates the time-course of key ECM events in relation to the classical clinical phases of ALI (exudative, proliferative, and fibrotic). In the early exudative phase, injury to alveolar epithelial and endothelial cells leads to basement membrane degradation, driven predominantly by protease activation and inflammatory signaling. Concurrently, increased vascular permeability promotes plasma protein leakage and hyaline membrane formation, characterized by fibrin-rich provisional matrix deposition along the alveolar surface. As ALI progresses into the proliferative phase, ECM remodeling becomes more heterogeneous, with overlapping matrix degradation and early collagen synthesis. In the fibrotic phase, persistent injury and dysregulated repair result in excessive deposition of fibrillar collagen, leading to matrix stiffening and architectural distortion. Red arrows denote carbon dioxide (CO_2_), and blue arrows denote oxygen (O_2_). The thickness of the arrows is proportional to the reduction in gas exchange. MMPs: Matrix metalloproteinases; DAMPs: Damage-associated molecular patterns; Created in BioRender. Fy, X. (2025) https://BioRender.com/v8zewb9.

The pathological process of ALI can be divided into three stages: the acute or exudative phase (days 1–6), characterized by disruption of the alveolar–capillary barrier, formation of hyaline membranes, and infiltration of neutrophils and other inflammatory cells; the proliferative phase (days 7–14), marked by the proliferation of type II alveolar epithelial cells and fibroblasts, accompanied by early collagen deposition; and the fibrotic phase (after day 14), where continued activation of fibroblasts leads to abnormal ECM deposition and remodeling of the lung structure [8]. While this staging remains clinically useful, it underestimates the immediacy and biological complexity of ECM dynamics. Increasing evidence suggests that ECM remodeling does not merely emerge during the late fibrotic phase but is initiated within hours of injury and evolves continuously across all stages of ALI [27,42]. Thus, reconsidering classical ALI staging through the lens of ECM remodeling reveals a temporally overlapping, rather than sequential, process in which matrix turnover is deeply integrated with immune activation and barrier dysfunction (Figure 1).

### 3.2. Evidence for ECM Synthesis–Degradation Disequilibrium

Contrary to the traditional view that matrix deposition is a hallmark of the fibrotic phase, several clinical observations demonstrate that active ECM remodeling begins during the initial inflammatory stage. Collagen type III N-terminal propeptide has been detected in bronchoalveolar lavage fluid (BALF) and serum of ALI/ARDS patients early after injury, indicating the onset of type III collagen synthesis during the exudative phase [53,54,55]. Simultaneously, ECM degradation is markedly accelerated. Elevated levels of matrix metalloproteinases (MMPs), elastin degradation products, and the fibronectin EDA splice variant (A(+)FN) are consistently observed in BALF and plasma samples from ALI patients [56,57,58,59,60]. In coronavirus disease 2019 (COVID-19)-related acute lung injury, proteomic analyses further reveal profound reductions in core ECM constituents—including fibrillar collagens, glycoproteins, and proteoglycans [61]. Together, these findings indicate that early ECM remodeling in ALI is characterized by an active process of both synthesis and degradation, forming a pathological remodeling microenvironment.

Importantly, this early imbalance between ECM synthesis and degradation involves not only fibrillar proteins but also HSPGs, a regulatory ECM component often overlooked in discussions centered on MMPs [62]. Beyond collagen XVIII, transmembrane and matrix-associated HSPGs—including syndecans, glypicans, and perlecan—stabilize the alveolar and endothelial basement membranes and sequester cytokines and chemokines via their heparan sulfate glycosaminoglycan side chains [63,64]. During the initial inflammatory phase of ALI, increased vascular permeability and cytokine signaling rapidly activate heparanase, the sole endoglycosidase in humans and other mammals capable of degrading heparan sulfate [63,65,66]. Loss of heparan sulfate represents an upstream event in ECM remodeling that exposes previously shielded core proteins, thereby enabling subsequent MMP-mediated proteolysis [62]. In parallel, heparanase-mediated degradation of endothelial heparan sulfate facilitates leukocyte transendothelial migration and releases ECM-bound inflammatory mediators, amplifying tissue injury [67,68]. Consistent with these mechanisms, increased circulating heparan sulfate degradation activity and elevated heparanase expression have been reported in patients with sepsis-associated ALI and in human lung tissues exhibiting diffuse alveolar damage [65]. Collectively, these observations place HSPGs and heparanase at the intersection of inflammatory signaling, leukocyte recruitment, and downstream ECM breakdown, reinforcing the concept that ECM synthesis–degradation disequilibrium is established at the earliest stages of lung injury.

### 3.3. ECM as an Active Signaling in Early Lung Injury

The biological relevance of early ECM remodeling extends far beyond structural disruption. ECM breakdown generates bioactive fragments—matrikines, that function as endogenous danger signals capable of engaging pattern recognition receptors (PRRs) on immune cells [69,70,71]. In addition, when functional sites of an ECM fragment originate from cryptic domains within the parent matrix rather than exogenously created sites, such fragments are also referred to as matricryptins [72]. Hyaluronan oligosaccharides and elastin-derived matrikines, for instance, accumulate during early injury and act as damage-associated molecular patterns (DAMPs) that amplify inflammatory signaling [26,73,74,75,76]. These fragments initiate and perpetuate a feed-forward cascade: initial injury triggers ECM degradation; degradation products activate immune cells; activated immune cells release additional proteases and inflammatory mediators, which further intensify ECM turnover. This self-reinforcing loop delays alveolar–capillary barrier repair, sustains inflammation, and biases the lung toward aberrant fibroproliferation. Thus, the ECM in early ALI functions not as a passive scaffold but as a dynamic signaling organ that integrates injury cues, modulates immune activity, and influences the trajectory toward resolution or fibrosis.

## 4. Immune Dysregulation as a Driver of ECM Remodeling

### 4.1. Immune Activation and Amplification

Immune activation constitutes the earliest and most decisive event shaping ECM remodeling in ALI. Upon exposure to pathogens, resident alveolar macrophages detect pathogen-associated molecular patterns (PAMPs) or DAMPs via PRRs, initiating the release of pro-inflammatory cytokines (interleukin-1β, Tumour necrosis factor-alpha), chemokines, and reactive oxygen species (ROS) [77,78]. These mediators activate endothelial cells, upregulate intercellular cell adhesion molecule-1 (ICAM-1) and vascular cell adhesion molecule 1 (VCAM-1) expression, and orchestrate rapid recruitment of neutrophils from the circulation. Neutrophils entering the alveolar and interstitial compartments serve as dominant early effectors of ECM injury. Through the release of ROS, neutrophil elastase, and MMPs, as well as the formation of neutrophil extracellular traps (NETs) that locally concentrate and activate proteases, they directly degrade basement membrane components and disrupt the structural integrity of the alveolar septa [79,80].

Importantly, dysregulated macrophage and neutrophil activation not only sustains tissue injury but also converts ECM degradation into a secondary inflammatory signal. Proteolytic cleavage of collagen, laminin, and elastin generates ECM fragments that function as endogenous DAMPs, capable of activating PRRs such as Toll-like receptor 2 (TLR2) and Toll-like receptor 4 (TLR4) on macrophages and neutrophils [26]. This establishes a self-propagating amplification loop in which immune activation drives ECM degradation, and ECM-derived signals in turn reinforce immune activation.

### 4.2. Cytokine Networks as Modulators of ECM Remodeling

Pro-inflammatory cytokines act as central regulators of ECM turnover. interleukin-1β (IL-1β) and Tumour necrosis factor-alpha(TNF-α) induce transcriptional upregulation of multiple matrix metalloproteinases (*MMP-1*, *MMP-8*, *MMP-9*) while suppressing tissue inhibitors of metalloproteinases (*TIMP-1/2*), thus tipping the balance toward excessive ECM degradation [81,82,83,84,85,86,87,88,89,90]. This imbalance accelerates the breakdown of collagen, laminin, and elastin within the alveolar basement membrane. In contrast, Interleukin-6 (IL-6) and transforming growth factor-β (TGF-β) promote ECM accumulation via fibroblast activation and enhanced collagen and laminin synthesis, contributing to matrix stiffening and myofibroblast differentiation [91]. The coexistence of these opposing signals generates a dysregulated remodeling microenvironment characterized by concurrent degradation and pathological deposition rather than coordinated repair.

Clinical evidence underscores the pathological relevance of cytokine-driven protease imbalance. In a prospective cohort, altered serum TIMP-1/MMP-9 ratios correlated strongly with disease severity and early mortality in ALI patients, highlighting cytokine-mediated ECM dysregulation as both a mechanistic driver and a clinically informative biomarker [92].

### 4.3. Immune-Driven Protease Release and ECM Degradation

In addition to cytokines, immune cells act as direct executors of ECM degradation by releasing a spectrum of proteolytic enzymes. Neutrophils secrete elastase, which readily degrades elastin, fibronectin, and interstitial collagens [93]. Activated macrophages produce MMP-9 and MMP-12, whose activities contribute to basement-membrane disruption and loss of alveolar structural integrity [94,95]. Excess proteolysis liberates bioactive matrikines that activate PRRs, including TLR4, on macrophages and neutrophils, thereby amplifying inflammatory cytokine release and protease production [26]. Thus, ECM degradation functions not merely as a consequence of immune activation but as an active amplifier of immune dysregulation. For instance, low-molecular-weight fragments of hyaluronan and oxidatively modified ECM components engage TLR4-dependent NF-κB signaling in macrophages, driving secondary production of IL-1β and TNF-α and reinforcing protease-mediated matrix degradation [73,96].

Beyond facilitating matrix degradation, heparanase plays a pivotal role in immune cell trafficking during ALI [65]. Interendothelial migration of leukocytes from the circulation into inflamed lung tissue requires localized degradation of endothelial basement membrane heparan sulfate. Activated neutrophils and monocytes express and secrete heparanase, enabling them to breach the heparan sulfate-rich endothelial glycocalyx and basement membrane, thereby gaining access to the interstitial and alveolar compartments [62,97,98]. While facilitating leukocyte recruitment, excessive heparanase activity disrupts endothelial barrier integrity, exacerbates vascular leak, and accelerates ECM destabilization. In this manner, heparanase integrates immune activation with progressive matrix injury, coupling leukocyte infiltration to worsening structural damage.

Together, immune activation in ALI initiates a highly interconnected cascade wherein cytokines, ROS, and proteases jointly disrupt ECM homeostasis; in turn, ECM degradation products function as bioactive DAMPs that amplify immune activation, establishing a self-sustaining injury circuit.

## 5. Oxidative Stress and ECM Structural Damage

### 5.1. ROS-Mediated ECM Oxidation and Fragmentation

A central pathological driver in ALI is the imbalance between excessive ROS production and the capacity of the endogenous antioxidant defense system [3]. This redox dysregulation directly exacerbates structural and functional impairment of the alveolar-capillary barrier [3]. ROS exerts both direct and indirect effects on ECM homeostasis during ALI. Directly, ROS oxidize amino acid residues such as proline and lysine within collagen, HA, and elastin, thereby disrupting their tertiary structure and reducing the mechanical stability of the ECM scaffold [99,100,101,102,103,104]. Oxidative modification of ECM proteins further alters their binding affinities for integrins and growth factors, impairing downstream signaling cascades critical for tissue repair [105]. Indirectly, ROS upregulate MMP-2, MMP-9, and MMP-13 expression, while simultaneously suppressing TIMPs [106,107,108], thus amplifying ECM degradation. Moreover, ROS impair ECM synthesis by damaging the endoplasmic reticulum in fibroblasts, leading to reduced production of collagen [109].

### 5.2. Mitochondrial Dysfunction as an Upstream Driver of ECM Injury

Beyond serving as a source of ROS, mitochondrial dysfunction functions as a central signaling hub linking cellular stress to inflammatory activation and ECM remodeling. In injured alveolar epithelial cells and macrophages, pulmonary irritants induce mitochondrial damage, leading to cytosolic release of mitochondrial DAMPs, including mitochondrial ROS (mtROS), mitochondrial DNA (mtDNA), cardiolipin, and cytochrome c [110]. mtROS acts as a proximal inflammatory trigger by inducing mitochondrial damage that leads to the release of NLRP3-activating signals, leading to caspase-1 activation and subsequent maturation and release of IL-1β [3,111]. IL-1β serves as a critical molecular bridge between mitochondrial dysfunction and ECM remodeling by inducing transcription and activation of matrix metalloproteinases, including MMP-1, MMP-9, and MMP-13, thereby accelerating collagen and elastin degradation within the alveolar ECM [112,113,114,115,116]. In parallel, mtROS promotes nuclear translocation of NF-κB p65, further amplifying pro-inflammatory gene expression. mtDNA released into the cytosol functions as a ligand for TLR9, reinforcing NLRP3 inflammasome signaling [117]. Additional mitochondrial-derived cues, including cardiolipin and Ca^2+^ flux, directly activate NLRP3, while extracellular ATP released from injured cells engages the purinergic receptor P2X7, facilitating NLRP3 mitochondrial localization and further enhancing mtROS production [118,119].

Mitochondrial damage also promotes inflammatory cell recruitment. Release of N-formylated peptides activates formyl peptide receptor 1 (FPR1), while increased CXCL8 production further enhances neutrophil recruitment and inflammatory amplification [120,121]. Recruited neutrophils contribute to downstream ECM injury through the release of proteases and reactive oxygen species, reinforcing matrix degradation initiated by mitochondrial stress.

Defective mitophagy exacerbates this cascade by permitting accumulation of dysfunctional mitochondria, sustaining mtROS generation, and prolonging inflammasome signaling. In parallel, mitochondrial bioenergetic failure impairs ATP-dependent cytoskeletal organization and cell–matrix adhesion, rendering epithelial and endothelial cells more susceptible to detachment and death. Pyroptotic and apoptotic cell death associated with mitochondrial dysfunction releases additional DAMPs, including HMGB1 and oxidized phospholipids, which further enhance neutrophil recruitment and protease release [122,123,124].

Collectively, mitochondrial injury establishes a self-perpetuating feed-forward loop in which mtROS–NLRP3–IL-1β signaling drives MMP-mediated ECM degradation, while ECM damage and inflammatory cell recruitment generate secondary danger signals that reinforce inflammasome activation, neutrophilic inflammation, and progressive alveolar barrier disruption in ALI.

### 5.3. Surfactant Disruption and Secondary ECM Instability

Inactivation of pulmonary surfactant is a characteristic feature of ALI and predisposes the alveolar microenvironment to ECM injury by disrupting biomechanical homeostasis and immune regulation. Loss of surfactant increases alveolar surface tension, exposing epithelial cells to exaggerated mechanical stretch during respiration, which predisposes the alveolar–capillary barrier to structural damage [3,125,126]. Beyond its biophysical role, surfactant exerts critical control over innate immune signaling within the alveolar microenvironment. Among surfactant-associated proteins, surfactant protein D (SP-D) functions as a key immunomodulatory molecule that restrains TLR4-dependent inflammatory activation. SP-D interacts with the TLR4 complex via its carbohydrate recognition domain, thereby suppressing downstream PI3K/Akt and NF-κB signaling and limiting production of pro-inflammatory cytokines such as IL-1β and TNF-α [127,128,129]. Loss or inactivation of SP-D removes this inhibitory checkpoint, permitting exaggerated TLR4 signaling and sustained innate immune activation [127]. This inflammatory signaling cascade directly impacts ECM homeostasis. TLR4–NF-κB-dependent cytokine release promotes transcription and activation of matrix metalloproteinases, particularly MMP-13, leading to accelerated degradation of collagen and other structural ECM components. Consistent with this mechanism, SP-D has been shown to suppress MMP-13 expression and preserve collagen integrity, indicating that surfactant dysfunction facilitates ECM breakdown through defined immune signaling pathways rather than passive mechanical effects alone [127,130].

Collectively, oxidative stress in ALI drives ECM injury through a hierarchical and mechanistically interconnected network rather than through nonspecific oxidative damage alone. Together, these processes establish self-sustaining feed-forward loops in which oxidative stress, mitochondrial dysfunction, immune activation, and ECM degradation are mutually reinforcing. This reciprocal coupling of redox imbalance, inflammatory signaling, and matrix remodeling provides a mechanistic framework for understanding progressive alveolar–capillary barrier failure in ALI and highlights key molecular nodes—such as mtROS–NLRP3–IL-1β signaling and SP-D–TLR4 regulation—as potential therapeutic targets.

## 6. Coagulation–Fibrinolysis Imbalance and Matrix–Immune Coupling

### 6.1. Procoagulant Activation and Provisional Matrix Formation

The coagulation system is rapidly activated in ALI. Endotoxin- and cytokine-stimulated endothelial cells express tissue factor, initiating the extrinsic coagulation pathway and promoting fibrin deposition within the alveolar and microvascular compartments [131]. Platelet–neutrophil interactions facilitate NETs formation and immunothrombosis [132], generating a provisional ECM scaffold rich in fibrin and fibronectin. Beyond serving as a passive scaffold, the fibrin–fibronectin-rich provisional matrix actively regulates immune cell behavior. Fibrin engages integrins such as α_M_β_2_ on neutrophils and macrophages, enhancing leukocyte adhesion, retention, and survival within the injured alveolar space [133]. Fibronectin further amplifies inflammatory signaling by promoting macrophage activation and fibroblast migration [134]. While transient matrix deposition supports early host defense, excessive and persistent accumulation drives fibroblast activation, myofibroblast differentiation, and collagen synthesis, thereby linking coagulation activation directly to pathological ECM remodeling and fibrotic progression [135,136,137,138]. Conversely, ECM components reciprocally modulate coagulation cascades. Collagen types I and III activate platelets via glycoprotein VI, while fibronectin binds coagulation factor XIII, promoting fibrin cross-linking and matrix stabilization [139,140,141]. This bidirectional interaction establishes a feed-forward loop in which coagulation and ECM remodeling mutually reinforce inflammatory amplification.

### 6.2. Impaired Fibrinolysis and Pro-Fibrotic Signaling

In parallel with procoagulant activation, ALI is characterized by profound suppression of fibrinolysis. Lung tissue and bronchoalveolar lavage fluid exhibit elevated expression of plasminogen activator inhibitor-1 (PAI-1), resulting in impaired plasmin generation and defective fibrin clearance [142]. Additionally, levels of activated protein C (APC), a critical anticoagulant and cytoprotective mediator, are markedly reduced in ALI patients. Both elevated PAI-1 and reduced APC levels correlate strongly with increased mortality, underscoring the clinical relevance of coagulation–fibrinolysis imbalance [143]. Importantly, PAI-1 exerts profibrotic effects independent of its antifibrinolytic function. By binding to vitronectin, an ECM glycoprotein, PAI-1 enhances integrin-mediated fibroblast adhesion and promotes differentiation into myofibroblasts, thereby accelerating ECM deposition and matrix stiffening [144,145,146]. Additionally, by inhibiting urokinase-type plasminogen activator (uPA), PAI-1 impairs the generation of plasmin, which is required for the proteolytic activation of latent TGF-β, a master regulator of fibrosis. This further disrupts normal repair and accelerates pathological ECM deposition [147,148,149]. Collectively, impaired fibrinolysis transforms the ECM into a fibrin-dominated, inflammation-permissive niche that sustains immune activation and drives fibrotic remodeling.

**Figure 2:**
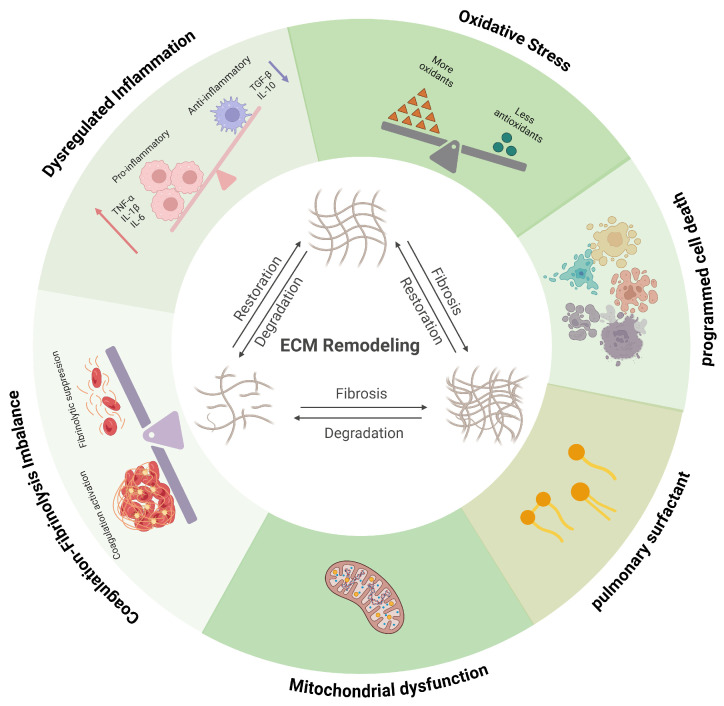
Schematic representation of the pathological progression of ALI and its association with ECM remodeling. Immune dysregulation, oxidative stress, mitochondrial dysfunction, surfactant disruption, programmed cell death, and coagulation imbalance interact dynamically with ECM remodeling during ALI, forming tightly coupled and self-reinforcing pathogenic networks. Red arrows indicate increased protein levels of tumour necrosis factor-alpha (TNF-α), interleukin-1β (IL-1β), and interleukin-6 (IL-6); blue arrows indicate decreased protein levels of transforming growth factor-β (TGF-β) and interleukin-6 (IL-10). Created in BioRender. Fy, X. (2025) https://BioRender.com/o3pyrzl.

Collectively, immune dysregulation, oxidative stress, mitochondrial dysfunction, surfactant disruption, programmed cell death, and coagulation imbalance converge to drive ECM remodeling in ALI through tightly coupled signaling networks. Immune activation and redox stress initiate ECM degradation via protease release and mtROS–NLRP3–IL-1β–MMP signaling, while matrix-derived DAMPs, provisional fibrin matrices, and impaired fibrinolysis reciprocally amplify inflammatory and coagulative responses. These bidirectional feedback loops transform the ECM from a structural scaffold into an active signaling platform that sustains leukocyte recruitment, protease activity, and fibroproliferative remodeling. Together, these interconnected pathways create a self-perpetuating loop of inflammation, matrix degradation, and fibroproliferation, positioning ALI as a disease fundamentally rooted in reciprocal immune–ECM crosstalk (Figure 2).

## 7. Biochemical Remodeling of the ECM and Immune Regulation

### 7.1. Collagen-Derived Matrikines and Immune Regulation

Collagen, the most abundant ECM component in lung tissue—particularly types I, III, and IV—plays a fundamental role in maintaining pulmonary structural integrity and homeostasis. During ALI, collagen undergoes dynamic cycles of degradation and synthesis, and its cleavage fragments are released into the alveolar space, airways, and circulation. Among the best-characterized collagen-derived matrikines are proline–glycine–proline (PGP) and its acetylated form (AcPGP). PGP is generated by sequential enzymatic cleavage of collagen through the concerted action of MMP-9/MMP-8 and prolyl endopeptidase (PE). Accumulated PGP acts as a potent neutrophil chemoattractant, amplifying inflammation and sustaining protease–antiprotease imbalance [150].

PGP functions as a matrikine by activating CXCR1/2 signaling pathways on neutrophils, thereby establishing a prototypical ECM–immune feedback loop: neutrophil-derived proteases generate PGP, which in turn recruits additional neutrophils and amplifies proteolytic ECM injury. However, PGP can be degraded by leukotriene A4 hydrolase (LTA4H), representing a secondary anti-inflammatory mechanism thatlimits sustained inflammation [151]. N-terminal acetylation yields AcPGP, a degradation-resistant ligand that sustains CXCR1/2-mediated neutrophil recruitment and inflammatory activation [152,153]. In experimental ALI models, elevated PGP and AcPGP levels correlate with excessive neutrophil infiltration, endothelial barrier dysfunction, and aggravated lung injury [152,154,155,156].

Beyond PGP/AcPGP, other collagen-derived fragments also exert diverse immunomodulatory effects. For instance, a peptide fragment DGGRYY from type I collagen activates neutrophils by binding to lymphocyte function-associated antigen 1 (LFA-1) [157]. Not all collagen-derived matrikines are pro-inflammatory. The tripeptide glycyl–L-histidyl–L-lysine (GHK), derived from type I collagen and the matricellular protein SPARC (Secreted Protein Acidic and Rich in Cysteine), enhances pulmonary antioxidant enzyme activity, suppresses IL-6 and TNF-α expression, and limits neutrophil infiltration, thereby mitigating lung injury [158,159,160]. Likewise, tumstatin, generated by MMP-9–mediated cleavage of type IV collagen, inhibits eosinophil and lymphocyte infiltration, displaying potent anti-inflammatory properties [161,162,163].

In addition, endostatin, a type XVIII collagen–derived matrikine, exhibits multifaceted biological effects in the injured lung. Its combined actions on neutrophil chemotaxis, platelet aggregation, and endothelial barrier disruption suggest that endostatin may act as a critical mechanistic link among these cellular events in the pathogenesis of ARDS, bridging inflammatory and vascular dysfunction within the remodeled ECM microenvironment [164]. Clinically, circulating endostatin has been identified as an early biomarker of COVID-19 disease severity, underscoring its potential as both a diagnostic and mechanistic indicator of pulmonary vascular injury and dysregulated ECM remodeling [165]. These findings highlight collagen-derived matrikines as active signaling intermediates rather than inert byproducts of matrix degradation.

Overall, collagen degradation in ALI generates a diverse repertoire of matrikines that actively regulate immune and vascular responses rather than serving as inert byproducts of tissue injury. Pro-inflammatory fragments such as PGP/AcPGP establish self-amplifying neutrophilic feedback loops, whereas anti-inflammatory peptides, including GHK and tumstatin, counterbalance excessive inflammation, highlighting collagen-derived signals as key determinants of immune–ECM reciprocity and disease trajectory in ALI.

### 7.2. Elastin-Derived Matrikines and Immune Regulation

Elastin fibers are essential for maintaining lung compliance and elastic recoil. In chronic obstructive pulmonary disease (COPD), for instance, diminished alveolar elastic recoil is intimately linked to elastin fibre structural abnormalities [166]. Proteolytic degradation of elastin during lung injury generates bioactive fragments that exert chemotactic and immunomodulatory effects. Elastin-derived peptides of 10–50 kDa efficiently recruit monocytes and macrophages to sites of injury, thereby amplifying inflammatory responses [167,168]. The elastin-derived hexapeptide VGVAPG exhibits chemotactic activity toward monocytes, macrophages, and fibroblasts, promoting immune cell accumulation and fibroblast activation within injured lung tissue [169,170]. Through engagement of elastin-binding receptors, these matrikines further reinforce inflammatory cell recruitment and ECM remodeling, linking mechanical fiber breakdown to immune dysregulation.

### 7.3. Hyaluronan-Derived Matrikines and Immune Regulation

Under physiological conditions, hyaluronan (HA) exists predominantly as high-molecular-weight (HMW) polymers that contribute to tissue hydration and structural stability. During lung injury and inflammation, oxidative stress and hyaluronidase activity generate low-molecular-weight HA fragments that accumulate at sites of injury. The clearance of these fragments—via the receptor CD44—is considered important in mitigating lung injury [171]. HA fragments activate macrophages and dendritic cells through TLR2 and TLR4 signaling, inducing the release of chemokines and pro-inflammatory cytokines [172,173,174]. In contrast, endogenous HMW HA delivers protective signals that promote epithelial survival and tissue repair [175,176,177]. Thus, HA metabolism exemplifies a size-dependent immune switch, whereby ECM fragmentation converts a homeostatic structural component into a potent inflammatory signal that amplifies immune activation and matrix degradation.

### 7.4. Fibrin/Laminin-Derived Matrikines and Immune Regulation

Fibrin turnover is closely linked to inflammatory signaling and tissue repair. Human fibrin peptide B, generated during fibrinogen cleavage, recruits neutrophils and fibroblasts, thereby contributing to inflammatory amplification and matrix deposition [178]. Similarly, laminin cleavage by neutrophil elastase releases matrikines that promote neutrophil recruitment and exacerbate lung injury [179]. These findings underscore that provisional matrix components not only provide structural support but also actively generate immunoregulatory signals that reinforce inflammation and delay resolution.

### 7.5. Heparan Sulfate Proteoglycans as Cytokine Reservoirs and Immune Modulators

HSPGs organize immune signaling within the pulmonary ECM by binding cytokines, chemokines, and growth factors, including IL-8, CXCL1, VEGF, FGF, and TGF-β [63]. In ALI, heparanase-mediated cleavage of heparan sulfate chains releases ECM-bound cytokines into the alveolar and interstitial spaces, abruptly amplifying immune cell recruitment and activation [62]. This disruption of cytokine compartmentalization transforms the ECM from a regulatory reservoir into a driver of uncontrolled inflammation, reinforcing immune dysregulation and impairing coordinated repair responses.

Collectively, ECM degradation in ALI generates a broad spectrum of matrikines and liberated cytokines that actively instruct immune and vascular responses rather than representing passive byproducts of tissue injury. Through receptor-specific signaling and self-amplifying feedback loops, these ECM-derived signals couple matrix breakdown to sustained inflammation, immune cell recruitment, and dysregulated repair, thereby shaping disease progression and resolution (Table 1).

## 8. Physical Remodeling of the ECM and Mechanoregulation of Immunity

### 8.1. Mechanical Cues as Immune Modulators

Mechanical forces are increasingly recognized as major determinants of immune cell behavior. Immune cells patrolling the lung microenvironment rely not only on receptor–ligand interactions but also on mechanical sensing to regulate migration, activation, differentiation, and effector functions [180,181,182,183]. In the mechanically dynamic lung, ECM biophysical properties serve as a primary interface through which cells perceive stress and convert it into intracellular signaling [26,184]. ALI disrupts these properties via edema, matrix degradation, and abnormal deposition, thereby altering the mechanical landscape that shapes immune responses.

### 8.2. Mechanotransduction Pathways in Immune Cells

Integrin-based mechanotransduction is the most deeply characterized pathway through which immune cells sense ECM physical cues. Engagement of ECM ligands activates focal adhesion complexes, including FAK and Src, which regulate downstream signaling cascades such as NF-κB and MAPK [180]. For example, mechanical stretch drives macrophages toward an M1-like pro-inflammatory phenotype through FAK/NF-κB signaling [185]. Beyond integrins, the Piezo family of mechanosensitive ion channels, especially Piezo1, links mechanical ventilation to Ca^2+^ influx, cytoskeletal reorganization, barrier disruption, and cytokine release (TNF-α, IL-1β, and IL-6) [186,187]. These pathways position ECM mechanics as an upstream regulator of immune activation, linking physical matrix alterations directly to inflammatory signaling cascades.

### 8.3. Immune Cell Sensitivity to ECM Stiffness

Monocytes and macrophages exhibit pronounced sensitivity to matrix rigidity. Stiff substrates intensify LPS-induced cytokine release and promote dendritic cell-like differentiation, while soft matrices bias macrophages toward immunoregulatory phenotypes [188,189,190]. These stiffness-dependent transitions influence phagocytosis, metabolism, and migratory capacity, thereby modulating the inflammatory landscape in ALI [191,192,193]. Such mechanosensitive plasticity underscores the importance of ECM mechanics as a regulator of both immune activation and resolution.

### 8.4. ECM Stiffening and Inflammation as a Positive Feedback Loop

ECM stiffening and inflammation reinforce each other through tightly coupled feedback mechanisms. Inflammatory cells release large amounts of MMPs, intensifying ECM degradation and disrupting the alveolar basement membrane and intact ECM architecture. Mechanically stressed fibroblasts differentiate into myofibroblasts, producing excessive collagen and generating heterogeneous fibroblast subpopulations that perpetuate matrix deposition [194]. The resulting stiffened matrix further amplifies mechanotransduction-driven inflammatory signaling, establishing a self-perpetuating loop that sustains immune activation and promotes fibroproliferative remodeling.

### 8.5. Altered Basement Membrane Architecture in ALI

A hallmark manifestation of ECM physical remodeling is the destruction of the alveolar–capillary barrier’s basement membrane, which tightly regulates cell and molecular transit across the barrier. Its disruption is a defining event in ALI, directly causing dysregulated immune cell localization and pulmonary oedema formation [195,196]. Macrophages mediate efficient barrier penetration via MT1-MMP-dependent basement membrane degradation; alternatively, they may traverse interstitial ECM without protease activity by altering their morphology [197,198]. Activated neutrophils release MMPs and neutrophil elastase, degrade matrix protein “low expression regions” (LERs) within the basement membrane, expand these LERs, and carry laminin fragments, thereby enhancing their penetration capacity [199,200,201]. Furthermore, expansion of LERs has been shown to facilitate monocyte infiltration [202].

Basement membrane destruction permits uncontrolled immune cell entry into the alveolar space, where neutrophils and macrophages release high levels of proteases and pro-inflammatory cytokines (TNF-α, IL-1β). Accumulation of edema fluid and cellular debris further impairs gas exchange, culminating in hypoxemia and respiratory failure.

## 9. Novel Intervention Strategies Targeting ECM Remodeling

In ALI, pathological remodeling of the ECM is not merely a consequence of structural destruction but a pivotal driver of disease progression. Recent advances have highlighted multiple therapeutic approaches that specifically target the processes of ECM remodeling. An intriguing study demonstrated that inhalation of exogenous soluble ECM can attenuate acute lung injury [203,204,205], supporting the feasibility of ECM-based cytoprotective interventions. Such ECM-derived therapeutics may function as adjuncts to standard treatments rather than stand-alone modalities. Such ECM-derived therapeutics may serve as adjuncts or enhancers to conventional treatment strategies. This section summarizes emerging approaches aimed at inhibiting excessive ECM degradation, modulating immune–matrix interactions, and promoting restoration of ECM homeostasis.

Despite these encouraging advances, translating ECM-targeted interventions into consistent clinical benefit remains challenging. A major obstacle lies in the pronounced phenotypic heterogeneity of ALI/ARDS, encompassing distinct inflammatory, endothelial, epithelial, and fibroproliferative endotypes that are insufficiently captured by current preclinical models. Consequently, therapeutic strategies that demonstrate robust efficacy in experimental settings often fail to achieve uniform benefit in unselected patient populations. These considerations underscore the need for precision stratification and stage-aware therapeutic approaches when targeting ECM remodeling in clinical practice.

### 9.1. Interventions Targeting the Protease System

MMPs play central roles in the pathological progression of ALI [71,206]. The protease network has emerged as a key therapeutic target in ALI. Current approaches include three main categories of protease-directed interventions: small-molecule inhibitors, antibody-based biologics, and endogenous TIMPs [207,208]. Small-molecule inhibitors act by binding to catalytic or allosteric domains of MMPs to suppress their proteolytic activity; multiple preclinical studies have demonstrated their capacity to attenuate ECM degradation and reduce lung injury in ALI models [209,210,211,212,213,214,215]. Representative compounds such as CGS27023AM (CGS) have been shown to mitigate neutrophil infiltration and vascular leakage-associated pulmonary edema, underscoring their therapeutic promise [216]. Indirect inhibition of MMP-dependent pathways has also gained attention. The synthetic inhibitor Z-Pro-prolinal suppresses prolyl PE, thereby reducing PGP formation and dampening neutrophil chemotaxis [217]. Compared with small molecules, antibody-based inhibitors offer greater target specificity and reduced off-target toxicity [218,219,220,221]. TIMPs, as endogenous broad-spectrum metalloproteinase inhibitors, contribute physiologically relevant, multi-target regulatory effects [56,87,222,223,224,225,226,227]. While native TIMPs act as broad-spectrum MMP inhibitors, protein engineering enables the design of variants with refined specificity [228,229].

Overall, future optimization should emphasize isoform specificity, reduced systemic toxicity, and combinatorial strategies aligned with anti-inflammatory therapy.

### 9.2. Interventions Modulating Cell–Matrix Interactions

Macrophage polarization profoundly influences ECM remodeling. The S1P receptor antagonist JTE-013 restores M1/M2 macrophage balance, thereby promoting inflammation resolution and restraining pathological ECM deposition [230]. This immune-reprogramming strategy provides an indirect yet effective means of modulating ECM dynamics.

Biomaterial-based interventions also offer innovative approaches to ECM repair. In tissue-engineered heart valves, functionalization of decellularized ECM scaffolds with red blood cell membranes (RBCM) improved hemocompatibility, promoted M2-type macrophage polarization, and facilitated ECM remodeling [231]. Such biomaterial-guided immunomodulation may provide transferable principles for pulmonary tissue repair.

Fibroblasts, the primary source of collagen, remain central executors of ECM remodeling. Stage-specific targeting—suppressing fibroblast hyperactivation during early inflammation and modulating collagen synthesis during later fibrosis—represents a rational therapeutic strategy.

### 9.3. Stem Cell-Based Strategies for ECM Regeneration

Mesenchymal stem cells (MSCs) have attracted growing interest for ALI therapy [232,233]. By releasing paracrine factors and extracellular vesicles (EVs), MSCs exert anti-inflammatory, anti-apoptotic, antibacterial, and pro-angiogenic effects, enhancing bacterial clearance, alveolar fluid reabsorption, and repair of injured barriers [13]. These multimodal activities attenuate lung and systemic injury [234,235]. In experimental LPS-induced ALI, preconditioning MSCs with low-dose TGF-β1 or genetically modifying cytoskeletal dynamics enhanced ECM-related reparative functions [236,237]. MSCs also promote macrophage polarization toward reparative phenotypes essential for ECM homeostasis [238]. However, therapeutic efficacy is highly microenvironment-dependent. Endothelial cell-derived EVs in ALI/ARDS suppress IDH2 in MSCs, reducing α-ketoglutarate, inhibiting TET dioxygenase activity, and impairing MSC repair potential [239].

Overall, MSC-based therapy provides an integrative solution for ALI via multiple molecular and cellular pathways. Yet, its efficacy is tightly governed by the local microenvironment. Deeper exploration of MSC–microenvironment interactions, particularly in overcoming metabolic and epigenetic suppression, will be critical for developing more effective ECM-regenerative and lung-repair strategies.

### 9.4. Nanotechnology-Enabled Targeted Delivery Systems

Nanotechnology enables microenvironment-responsive therapeutic delivery for ALI. A GPQ-modified nanoplatform responsive to MMP-9 (GPQ-EL-DNP) releases DNP locally in fibrotic regions, inducing controlled thermal modulation and altering collagen architecture to reduce ECM stiffness [240]. Another system, RGD-modified liposomes encapsulating methylprednisolone sodium succinate (MPSL-cRGD), competitively blocks αvβ6/αvβ5 integrins and alleviates vascular hyperpermeability [241]. Macrophage-targeted nanotherapies further allow for selective modulation of pro-inflammatory M1 cells with minimal systemic exposure [242]. Collectively, ECM-responsive nanocarriers offer spatiotemporally controlled, precision therapeutic delivery for ALI.

### 9.5. Multi-Target Modulation by Natural Compounds

Natural products often exert multi-node regulatory effects, advantageous for complex conditions such as ALI. Harpagide inhibits HIF-1α and activates Nrf2/HO-1, thereby reducing ECM deposition and oxidative injury [243]. PNSC5325 decreases inflammatory cytokines and MMP activity, mitigating early ECM over-degradation [244]. These studies collectively indicate that natural compounds exert synergistic regulation over multiple pathogenic nodes, providing a multi-target therapeutic strategy for ALI. Compared with single-target drugs, such pleiotropic agents show superior potential in maintaining ECM homeostasis and thus represent promising candidates for novel pharmacological development.

Together, these emerging therapeutic strategies underscore the conceptual shift toward viewing the ECM not simply as a structural scaffold but as a dynamic and regulatable biological system. Targeting ECM remodeling at the molecular, cellular, and microenvironmental levels holds substantial promise for improving outcomes in ALI, paving the way for next-generation interventions that integrate immune regulation, matrix preservation, and tissue repair. It is also important to recognize that advanced life-support modalities, such as extracorporeal membrane oxygenation (ECMO), may exert complex and sometimes paradoxical effects on lung integrity and ECM homeostasis [245]. While often lifesaving, extracorporeal circulation can amplify systemic inflammatory responses through blood–artificial surface interactions, as highlighted in large-animal studies, and may thereby influence ECM injury and remodeling [246]. This duality underscores the importance of considering supportive technologies within an integrated framework that balances respiratory support with preservation of matrix integrity.

## 10. Conclusions

The intricate interplay between ECM remodeling and immune responses constitutes a dynamic and interdependent network in ALI. However, distinguishing which ECM alterations act as causative drivers versus secondary consequences of tissue injury remains a major challenge. Historically, research has emphasized ECM deposition in fibrotic disorders as a terminal manifestation. Increasing evidence now indicates that ECM degradation, mediated primarily by proteases such as MMP-8, MMP-9, and neutrophil elastase, is equally critical in disease progression and closely linked to inflammatory storms. Notably, early ECM remodeling may precede cytokine surges and actively exacerbate inflammation [247]. Proteolytic degradation of ECM releases chemotactic matrikines that recruit additional immune cells; concurrently, remodeled ECM provides physical conduits for immune cell migration, together forming a self-amplifying inflammatory loop. Future investigations should leverage cell-specific gene-editing models—e.g., conditional deletion of ECM components or their receptors in fibroblasts or myeloid cells—combined with advanced live imaging to dynamically and causally dissect the functional roles of specific ECM elements during ALI progression.

Significant heterogeneity among ALI patients [4] and the limited translational fidelity of current animal models further complicate clinical application. Because ECM proteins are poorly soluble and structurally integrated into the lung scaffold, direct sampling during disease is technically challenging. Most studies rely on circulating biomarkers or bronchoalveolar lavage analyses, which restrict mechanistic interpretation. To overcome these limitations, integration of single-cell transcriptomics, spatial omics, and mass spectrometry imaging should be employed to delineate ECM remodeling patterns and associate them with clinical outcomes such as fibrotic propensity or relapse risk, thereby establishing a foundation for precision stratification and targeted therapy.

The ECM functions not as a static scaffold but as a dynamic regulatory network defined by both its physical (e.g., stiffness) and chemical (e.g., growth-factor sequestration) properties. Yet, its systemic regulatory principles remain poorly understood. Recent computational models based on spring-network simulations revealed that critical stiffness-percolation thresholds and initial damage topology (e.g., cluster coefficient and inter-cluster distance) determine tissue bifurcation toward healing or fibrosis [248]. To capture these dynamics, integrative approaches combining bioinformatics, modeling, and advanced 3D air–liquid interface cultures are required to elucidate how specific ECM attributes synergize or antagonize to shape immune cell responses and to identify nodal regulators within the network.

Importantly, ECM remodeling in ALI displays temporal duality: in the early phase, excessive degradation accelerates mediator diffusion and immune cell infiltration, whereas in the late phase, progressive deposition hampers functional regeneration. These stages overlap and interact, rather than occurring sequentially [27]. This phase-specific functional transition underscores the necessity of precise disease-stage delineation for effective intervention. Determining optimal therapeutic windows and developing adaptive, stage-responsive interventions are therefore imperative. Future strategies should aim to identify phase-specific biomarkers and design intelligent drug-delivery platforms that dynamically respond to disease progression, achieving truly spatiotemporal precision therapy.

In summary, the crosstalk between ECM remodeling and the immune system lies at the core of ALI pathogenesis. Future studies integrating multi-omics technologies, computational modeling, and genetic engineering will be essential to unravel the causal mechanisms of dynamic ECM remodeling. Concurrently, development of precise interventions capable of breaking the vicious cycle between inflammation and matrix remodeling, supported by cross-disciplinary collaboration and optimized preclinical models, will pave the way toward precision stratification and targeted treatment, ultimately improving ALI patient outcomes.

## Figures and Tables

**Table 1:** Summary of extracellular matrix (ECM)-derived matrikines and their pathophysiological impact in acute lung injury (ALI).

Parent Matrix	Matrikines	Protease	Cellular Effects	References
Collagen	Proline–Glycine–Proline (PGP)	MMP-9 MMP-8 PE	Promotes neutrophil chemotaxis; exacerbates protease imbalance; degraded by LTA4H to mitigate inflammation	[150,151,155]
	Acetylated PGP (AcPGP)	N-terminal acetylation of PGP	Resistant to LTA4H degradation; recruit neutrophils; ROS release; persistent inflammation; endothelial barrier dysfunction	[150,152,153,154,156]
Collagen I	DGGRYY peptide	-	Activates neutrophils	[157]
Collagen I and matricellular protein SPARC	GHK	MMP3	Enhances pulmonary antioxidant enzyme activity, suppresses IL-6 and TNF-α expression, and limits neutrophil infiltration	[158,159,160]
Collagen IV	Tumstatin	MMP9	Inhibits eosinophil and lymphocyte infiltration	[161,162,163]
Collagen XVIII	Endostatin	-	Regulates neutrophil chemotaxis, platelet aggregation, and endothelial barrier function	[164,165]
Elastin	10–50 kDa elastin fragments	Elastolytic enzymes	chemoattract monocytes and recruit monocyte–macrophages	[167,168]
	Val-Gly-Val-Ala-Pro-Gly	Elastolytic enzymes	Possesses chemotactic activity for monocytes, macrophages, and fibroblasts	[169,170]
HA	HA fragments (low molecular weight)	Hyaluronidases; ROS	Activates macrophages and dendritic cells;	[172,173,174]
Fibrin	Fibrin peptide B	Thrombin	Recruit neutrophils and fibroblasts	[178]
Laminin	Laminin fragments	Elastase	Recruit neutrophils	[179]

## Data Availability

No new data were created or analyzed in this study.

## Abbreviations

The following abbreviations are used in this manuscript:

ALI	acute lung injury
ECM	extracellular matrix
ARDS	acute respiratory distress syndrome
ICU	intensive care unit
AECC	American–European Consensus Conference
HA	hyaluronic acid
HSPGs	heparan sulfate proteoglycans
HFNO	high-flow nasal oxygen
BALF	bronchoalveolar lavage fluid
PPRs	pattern recognition receptors
PaO2/FIO2	partial pressure of arterial oxygen to fraction of inspired oxygen
DAMPs	damage-associated molecular patterns
PAMPs	pathogen-associated molecular patterns
IL-1β	interleukin-1β
NETs	neutrophil extracellular traps
TLR2	Toll-like receptor 2
FPR1	formyl peptide receptor 1
MMPs	multiple matrix metalloproteinases
mtROS	mitochondrial ROS
mtDNA	mitochondrial DNA
TIMPs	tissue inhibitors of metalloproteinases
TGF-β	transforming growth factor-β
ROS	reactive oxygen species
PAI-1	plasminogen activator inhibitor-1
uPA	urokinase-type plasminogen activator
APC	activated protein C
PGP	proline–glycine–proline
PE	prolyl endopeptidase
CPAP	continuous positive airway pressure
PEEP	positive end-expiratory pressure
LTA4H	leukotriene A4 hydrolase
LFA-1	lymphocyte function-associated antigen 1
GHK	glycyl–L-histidyl–L-lysine
SPARC	Secreted Protein Acidic and Rich in Cysteine
COPD	chronic obstructive pulmonary disease
VGVAPG	Val-Gly-Val-Ala-Pro-Gly
HMW	high molecular weight
LER	low expression regions
CGS	CGS27023AM
RBCM	red blood cell membranes
MSCs	Mesenchymal stem cells
EVs	extracellular vesicles
ECMO	extracorporeal membrane oxygenation
